# Treadmill walking during vocabulary encoding improves verbal long-term memory

**DOI:** 10.1186/1744-9081-10-24

**Published:** 2014-07-12

**Authors:** Maren Schmidt-Kassow, Nadine Zink, Julia Mock, Christian Thiel, Lutz Vogt, Cornelius Abel, Jochen Kaiser

**Affiliations:** 1 Institute of Medical Psychology, Goethe University, 60528 Frankfurt am Main, Germany; 2 Institute of Sport Sciences, Goethe University, Frankfurt am Main, Germany; 3 Max Planck Institute for Empirical Aesthetics, Frankfurt am Main, Germany

## Abstract

Moderate physical activity improves various cognitive functions, particularly when it is applied simultaneously to the cognitive task. In two psychoneuroendocrinological within-subject experiments, we investigated whether very low-intensity motor activity, i.e. walking, during foreign-language vocabulary encoding improves subsequent recall compared to encoding during physical rest. Furthermore, we examined the kinetics of brain-derived neurotrophic factor (BDNF) in serum and salivary cortisol. Previous research has associated both substances with memory performance.

In both experiments, subjects performed better when they were motorically active during encoding compared to being sedentary. BDNF in serum was unrelated to memory performance. In contrast we found a positive correlation between salivary cortisol concentration and the number of correctly recalled items. In summary, even very light physical activity during encoding is beneficial for subsequent recall.

## Introduction

Many anecdotal reports suggest that physical activity is beneficial for problem solving or memorizing of new information. For example, simultaneous promenading and reciting of text passages used to be a relevant part of the daily routine of ancient philosophers and monks. Pacing up and down the room during declarative learning and promenading while restructuring a complex problem has been reported to be helpful [1]. Hence, various field reports have indicated that simultaneous motor activity may enhance cognitive resources.

In the current article we distinguish between *physical exercise* and *motor activity*. We use the term *motor activity* to refer to any movements of very low intensity, i.e. finger tapping or promenading, while the term *exercise* is exclusively used for motor activity with an intensity of at least 46% VO2max which should induce changes in physical fitness and health [2]. With regard to the latter, there is increasing evidence that single bouts of physical exercise prior to a specific task improve cognitive functions. This has been shown for the speed of information processing [3-5], executive functions such as performance in Eriksen flanker task, Trail making test, or Stroop interference [6,7], enhanced cognitive flexibility [8], and mnemonic functions such as working memory [9,10], or long term memory [11-13]. There is evidence that the timing of exercise relative to a memory task modulates its effect. Labban and Etnier [14] showed that an acute exercise bout of moderate intensity prior to, but not after exposition to memory items resulted in a significantly better memorization compared to no exercise. Additionally, Salas et al. [12] found that walking (approximately moderate intensity, i.e. “the walking speed one would use when late to an appointment”, p. 509) prior to encoding but not prior to retrieval enhanced performance in a free recall task. Hence, acute exercise seems to be particularly beneficial for memory if temporally close to encoding. However, Winter et al. [13] found that although high-intensity exercise prior to learning results in faster vocabulary acquisition, neither high- nor low-intensity exercise led to enhanced vocabulary retention on the same day, after 1 week or after 8 months. In a similar vein, Coles and Tomporowski [11] found no positive effect of moderate-intensity bicycling prior to a free-recall test in comparison to sitting on a cycling ergometer or watching an educational documentary. With regard to simultaneous motor activity, a dual task effect might result in lower performance (for a comprehensive overview see [15]). Several studies have shown that low-intensity walking during memorizing impairs performance even in young and healthy adults (e.g. [15-18]; however see [19] for contrary effects in 9-year-olds). Results from our lab indicate the opposite: in two learning studies and an attention allocation experiment, low- and moderate-intensity simultaneous bicycling resulted in better performance compared to being sedentary [20-22]. In the current experimental series we investigated the effect of treadmill walking during vocabulary encoding on subsequent memory performance. We specifically aimed to investigate the effect of very low-intensity motor activity (approx. < 50% VO2max), i.e. far below the intensity levels typically investigated in studies on exercise and cognitive performance.

The search for endocrinological parameters mediating the effect of physical activity on mnemonic processes has focused on the brain-derived neurotrophic factor (BDNF) [13,23]. Several studies have indicated that BDNF levels are associated with cognitive processes such as memory (e.g., [24,25]), and that acute exercise of at least moderate intensity elevates both serum and plasma BDNF in humans (e.g., [13,26-31]). However, in our study on moderate-intensity bicycling during vocabulary encoding we did not find significant correlations between serum BDNF and learning performance [20], possibly because the motor activity was not sufficiently intense. This is in line with our observation that only high-, but not low-intensity bicycling results in a (transient) increase of BDNF in serum [29]. We measured BDNF in the first of the present two experiments because of its putative relevance for cognitive performance. However we did not expect it to be modulated by the present low-intensity motor activity.

Instead we tested in the second experiment an alternative explanation for the positive effect of exercise on cognition i.e., the arousal hypothesis (e.g. [32]). In a review, Tomporowski [33] found evidence for the relationship between cognition and physical arousal following the Yerkes-Dodson law [34]. Levitt & Gutin [35] reported faster reaction times for moderately increased heart rates, but slower reaction times at highly increased heart rates. According to a model by Kahneman [32] simultaneous low-intensity exercise increases the arousal level which in turn increases the resources available to perform a cognitive task. If on the other hand exercise withdraws resources necessary to perform the cognitive task, this should result in interference. In this case, the mental workload required by exercise would be too high to manage the cognitive task (cf. [36]). Furthermore physical exercise, at least at high intensity levels, has been reported to increase psycho-physiological stress which in turn increases the arousal level [37]. This affects salivary cortisol responses reflecting individual stress levels [38]. Hence, motor activity in combination with a cognitive task should increase the “participants’ arousal” and stress levels which may lead to deflections in salivary cortisol concentrations. Indeed Almela et al. [39] have shown that a higher stress-related cortisol response negatively influences memory performance in middle-aged women. However, others found beneficial effects of stress on memory (e.g. [40]). We investigated this issue in the current series of experiments.

Taken together, previous experiments on physical exercise and long-term memory as well as their endocrinological substrates have yielded inconsistent results. Our own data have indicated that physical activity during encoding is particularly beneficial for vocabulary learning, even for low to medium exercise intensity. In the current study, we further reduced exercise intensity resulting in an experimental condition where subjects were motorically active with minimal cardio-vascular load. Hence, we tested the anecdotal effect of promenading on cognition under controlled experimental conditions. In contrast to our previous studies [20,22], we tested equal numbers of male and female participants. Hence, we overcame a limitation of previous exercise studies testing either male or female subjects, or where the factor sex has not been parallelized (e.g. [10,13,14]). This is an important issue as previous studies have indicated that females may show better episodic memory functions than males [41,42]. Furthermore, we applied a within-subject design to assess the effect of slow treadmill walking on auditory vocabulary encoding, thus increasing the statistical power compared with between-subject designs. In the first experiment we measured BDNF in serum because of its putative relevance for cognitive performance, even though we did not expect it to be modulated by the present low-intensity activity. In a second experiment we followed the stress-arousal hypothesis and collected salivary cortisol.

We hypothesized that participants would show better learning performance during walking compared to the sedentary condition. However, previous studies showed that treadmill exercise may require cognitive resources, and hence subjects who are less familiar or confident on a treadmill might perform worse. In a recent review, Lambourne and Tomporowski [43] concluded that simultaneous cycling improved cognitive performance whereas simultaneous treadmill exercise deteriorated performance. This discrepancy was attributed to increased demands of treadmill exercise on keeping balance and on upper and lower limb coordination, possibly interfering with cognitive task demands. Hence, we asked the participants to rate how safe they felt on the treadmill to control for this issue. In line with our previous results, we expected BDNF in serum not to be elevated by walking. However, salivary cortisol should be increased in the walking condition compared to the sedentary condition due to increased arousal. Furthermore, performance in vocabulary tests should correlate positively with changes in salivary cortisol during encoding.

## Materials and methods

### Participants

In total 49 right-handed (as determined by the Edinburgh handedness inventory [44] monolingual German young and healthy subjects (aged between 18 and 30 years) participated in the current experimental series. Exclusion criteria were a history of psychiatric or neurological disorders, smoking, medication (except for contraceptives), obesity, and any knowledge of Polish or other Slavic languages (as participants were asked to learn Polish vocabulary). 18 individuals (9 females, mean age: 22.8 years, SD: 2.6, mean BMI: 23.2 kg/m^2^, SD: 2.9) took part in experiment 1. 31 subjects (16 females, mean age: 21.7 years, SD: 2.7, mean BMI 22.8 kg/m^2^, SD: 2.5) participated in experiment 2.

### Ethics statement

Both experiments are part of the project entitled “The influence of synchronous physical activity on brain plasticity and foreign language learning” which has been approved by the Ethics Committee of the Goethe University of Frankfurt Medical Faculty. It was conducted in accordance to the principles laid down in the Declaration of Helsinki. All subjects were informed about the aims of the study and gave their written consent.

### Procedure

Subjects were first screened in a pre-experimental evaluation session. After this session participants were asked to come to our laboratory for two experimental learning sessions. The experimental procedure was identical for experiment 1 and 2. The only difference was that we collected blood samples for BNDF analysis in experiment 1 and saliva samples for cortisol analysis in experiment 2.

### Pre-experimental screening

The participants completed several questionnaires. Their physical activity level was measured with the Freiburg Questionnaire of Physical Activity (FQPA; [45]. Furthermore, they had to indicate the number of foreign languages they had learned and the number of musical instruments they played. Additionally, we tested the candidates’ ability to memorize new vocabulary. They were asked to listen to 40 pseudowords followed by a German counterpart. All vocabulary pairs were presented via headphones. After thirty minutes during which participants continued to fill in the questionnaires listed above, subjects took part in a vocabulary test, i.e. they were presented the pseudowords and had to write down the associated German words.

Additionally, we asked participants to walk on a motor-driven treadmill (Tunturi, Turku, Finland) for about 20 minutes to determine their individual preferred walking rate. Each step was measured via a force sensitive resistor with a round, 0.5” diameter sensing area fixed at the participants left heel.

At the beginning of the study, all of the participants were instructed to avoid changes in their daily life including their physical activity level for the duration of the experiment (one week).

### Learning sessions

For the actual experiments, participants were asked to come twice to our laboratory. In one session subjects learned 40 Polish words (20 nouns and 20 verbs) while walking on the motor-driven treadmill at their preferred rate. Once a preferred speed had been chosen for each individual the walking rate and stimulation rate remained identical throughout the experiment. In a second session subjects learned another 40 Polish words while sitting in a canvas chair. The order of sessions was counterbalanced across subjects. We carefully controlled that the second session took place exactly 72 hours after the first, i.e. at the same time of day. Additionally, subjects participated in an online vocabulary test 24 hours after each learning session. We used a difficult auditory paired-associate learning paradigm to maximize the chances to observe an effect of walking on vocabulary encoding. Additionally, unimodal auditory stimulation prevents at its best from cheating on the recall test because participants have no information on how a respective word is spelled.

Each learning session lasted for 30 minutes during which participants listened to the Polish vocabulary twice. In addition 206 sinusoidal tones with a fundamental frequency of 250 Hz were presented at the beginning of each session, and 116 tones were presented in the middle of each session. This was done to familiarize participants with the new situation, and to signal a pause between the two learning blocks, respectively. Tones were presented at each participant's previously determined preferred walking rate. The loudness level was adjusted to their individual preference and kept constant across both learning sessions. The order of vocabulary pairs was randomized for each learning session and each participant. Stimuli were presented auditorily via headphones (Philips SHP 1900). Both Polish and German items were spoken by female native speakers of Polish and German, respectively, who had a phonetic-linguistic background. All stimuli were normalized to an intensity level of 75 dB using the software PRAAT. The stimulus onset asynchrony (SOA) of Polish–German vocabulary pairs was aligned to the perceptual center of a word [46], i.e. the vowel onset of the stressed syllable, and amounted to the preferred walking rate, i.e. to every fourth step. The average SOA was 2.8 s within a vocabulary pair corresponding to a word presentation at every fourth step and 8.4 s between vocabulary pairs, resulting in a pause of 12 steps before the next vocabulary pair has been presented. During this pause participants were asked to repeat the previously presented vocabulary pair loudly. We excluded action verbs to ensure that better performance in the walking session was not semantically induced [47].

In both experiments and both sessions, the individuals’ heart rate was constantly monitored with a chest strap (Polar S810, Polar, Büttelborn, Germany). In the walking session we additionally measured the participants’ walking rate with a force sensor and a customized microcontroller (®Arduino, http://www.arduino.cc) as described above.

The environment was kept constant across both learning conditions, i.e. both the *treadmill* and the *sedentary* session took place in the same room. At the end of each session, participants were asked to indicate how safe the felt on the treadmill and the quality of their last night's sleep. Answers were given on a 5-point Likert scale ranging from “low” to “high”. Furthermore, we asked the participants about their caffeine and alcohol consumption. They also had to indicate the number of sleeping hours in the previous 24 hours.

Twenty-four hours after each learning session subjects participated in a customized online vocabulary test. The access to the test was temporally limited to make sure that subjects adhered to their individual test time (+ - 1 hour). Here, they listened to all of the Polish vocabulary and were asked to enter the German translations in a response field. Response time was limited to 8 s for each item.

### Blood sampling

In experiment 1 two venous blood samples were taken from 11 out of 18 participants in each session. The other 7 participants declared that they felt uncomfortable with having blood taken. To avoid an additional stressor we refrained from blood samplings in these subjects. For the other participants, the first blood sample was collected after a ten-minute rest period. Hence, cardio-respiratory parameters were on a stable baseline level at the beginning of the learning period. The second blood sample was taken immediately after the particular intervention (learning while treadmill walking or sitting in a canvas chair) was finished.

### Analysis of BDNF serum concentrations

In each of the two blood samples, 4.5 ml of venous blood from the antecubital vein were collected with a clotted blood tube. Collection and analysis of blood samples was performed according to the following uniform protocol: all samples clotted within 30 minutes at a temperature of 21C. After the clotting period, samples were centrifuged for 10 minutes with 4800 rounds per minute using the Heraeus Labofuge 200 (Thermo Fisher Scientific, Germany). Immediately afterwards, serum was pipetted into separate SafeSeal micro tubes (Sarstedt, Nürnberg, Germany). Samples were stored at -30°C for one night and then transferred to a -80°C freezer for another three weeks. Then, BDNF levels in serum were measured using the Quantikine® Human BDNF Immunoassay from R&D Systems (Wiesbaden, Germany) with intra- and inter-assay precision of 6.2% and 8.1%, respectively.

The minimum detectable BDNF dose was less than 20 pg/mL, according to the manufacturer’s information. All samples were tested twice for reliability.

### Saliva sampling

In Experiment 2 two saliva samples were taken from all participants in each session. The first saliva sample was collected prior to each intervention. The second saliva sample was taken immediately after the particular intervention (learning while treadmill walking or sitting in a canvas chair) was finished.

### Analysis of cortisol concentrations

In the current study we used the “Salivette” (Sarstedt AG & Co., Nümbrecht) collection devices for collecting saliva. They consist of a cotton swab in a suspended insert which itself is placed in a centrifuge vessel.

Subjects were instructed to gently chew on the cotton swabs for one minute. Afterwards they return the saturated swab to the suspended insert without touching it. To reduce errors, subjects were not allowed to eat or drink anything else than water for one hour before the testing session started. Salivary samples were stored at -30°C until the biochemical analysis, which was performed by the Dresden Lab Service GmbH. Saliva samples were maximally stored for 47 days until they were analyzed. Samples were analyzed according to the protocol described in Nater et al. [48]. The concentration of free salivary cortisol was analyzed using a luminescence immunoassay (IBL, Hamburg, Germany) with intra- and inter-assay precision of 4.5% and 4.3%, respectively.

### Data analysis

For both experiments, performance in the vocabulary tests was compared using a 2*2 ANCOVA with the within-subject factor session (treadmill walking, sedentary), the between-subject factor sex, and the covariate “perceived safety on the treadmill”.

Experiment 1. To investigate the influence of BDNF on learning performance, we computed a 2*3*2 ANCOVA on BDNF in serum with the within-subject factors session (treadmill walking, sedentary) and time point (1,2), the between-subjects factor sex, and the covariate safety on the treadmill. Furthermore we calculated BDNF kinetics for learning (BDNF after learning minus BDNF at baseline) and computed correlations between BDNF at each time point as well as BDNF kinetics and vocabulary test performance for each experimental session.

Experiment 2. To investigate the influence of cortisol on learning performance we computed a 2*3*2 ANCOVA on salivary cortisol with the within-subject factors session (treadmill walking, sedentary) and time point (1,2), the between-subjects factor sex, and the covariate safety on the treadmill. Furthermore, we calculated the changes in cortisol levels during learning and computed correlations between cortisol kinetics and vocabulary test performance for each experimental session. One participant with cortisol levels deviating more than three standard deviations from the mean was excluded from the analysis.

## Results

### Participants

An overview of sample characteristics for experiments 1 and 2 is provided in Table 1. In both experiments, participants were moderately physically active and walked with a frequency of about 1.3 Hz. Presentation frequency and behavioral performance were not correlated, indicating that the individual presentation time did not affect retention performance (sedentary: r = -.02; p = .9; walking: r = -.09; p = .6).

**Table 1:** Sample characteristics in both experiments

	**Experiment 1**	**Experiment 2**
Freiburger Questionaire of Physical Activity (FQPA)	50 (31.8) MET-h/wk	50 (30) MET-h/wk
Preferred walking frequency	1.3 (0.17) Hz	1.36 (0.22) Hz
Foreign languages	2	2
Pre-experimental pseudoword learning	9.7 (4.6) out of 40 words	7 (4.4) out of 40 words
Heart rate treadmill/relaxed	100/69 (14.8/7.1) bpm	112/74 (10.4/7.1) bpm
Estimated walking intensity	52.9 (7.7) % HR_max_	58.7 (5.5) % HR_max_
Caffeine consumption	1.1 (1.6) cups	0.4 (0.8) cups
Alcohol consumption	0.5 (1.2) glasses	0.25 (0.6) glasses
Sleep quantity	7.1 (1.2) h	7.5 (1.1) h
Sleep quality	“good”	“good”

Mean heart rate (see Table 1) differed significantly between walking and being sedentary in both experiments according to a paired-samples *t*-test (Experiment 1: t (17) = 10.3; p < .001; d = 2.81, Experiment 2: t (28) = 18.7; p < .001; d = 3.7). In both experiments, walking intensity as estimated by heart rate [49] was very light according to the guidelines of the American College of Sports Medicine [2]. In both experiments, participants did not differ between sessions in their caffeine or alcohol consumption nor in their sleep quantity or quality (see Table 1).

### Experimental performance

**Figure 1:**
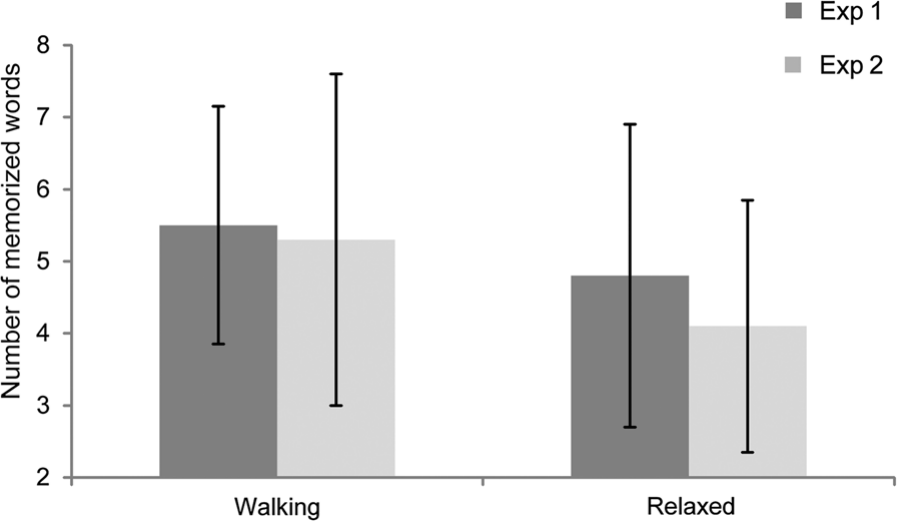
**Vocabulary test performance for each condition and experiment.** Experiment 1 = dark grey; Experiment 2 = light grey. Error bars indicate standard deviations.

For Experiment 1, the omnibus ANCOVA revealed a main effect of session (see Figure 1; F (1,15) = 6.98, p = .02, η_p_^2^ = .318; mean treadmill walking: 5.5 (SD: 3.3) words; sedentary: 4.8 (SD: 4.2) words) and an interaction of day of session x perceived safety on the treadmill (F (1,15) = 12.3, p < .01, η_p_^2^ = .451). There was no interaction with sex.

**Figure 2:**
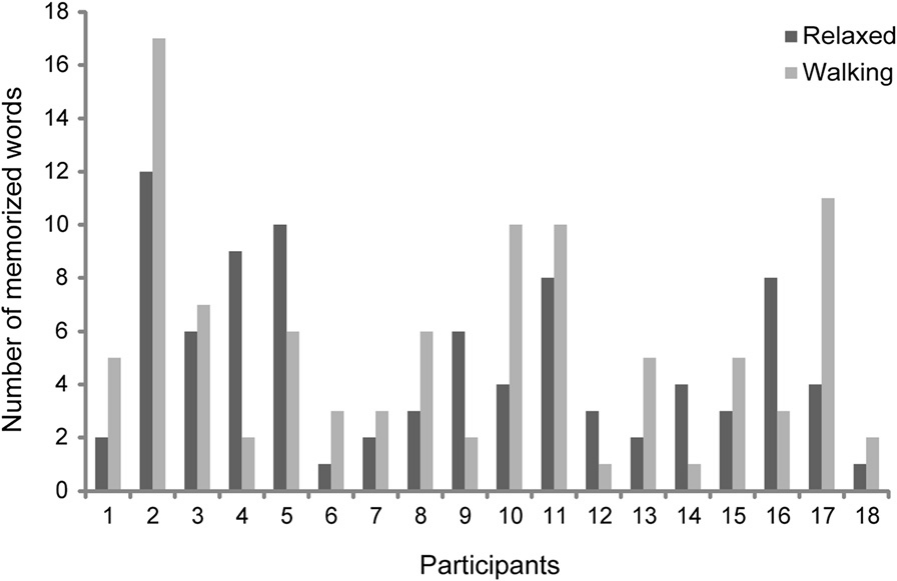
**Vocabulary test performance for each participant in Experiment 1.** Relaxed = dark grey; Walking = light grey.

**Figure 3:**
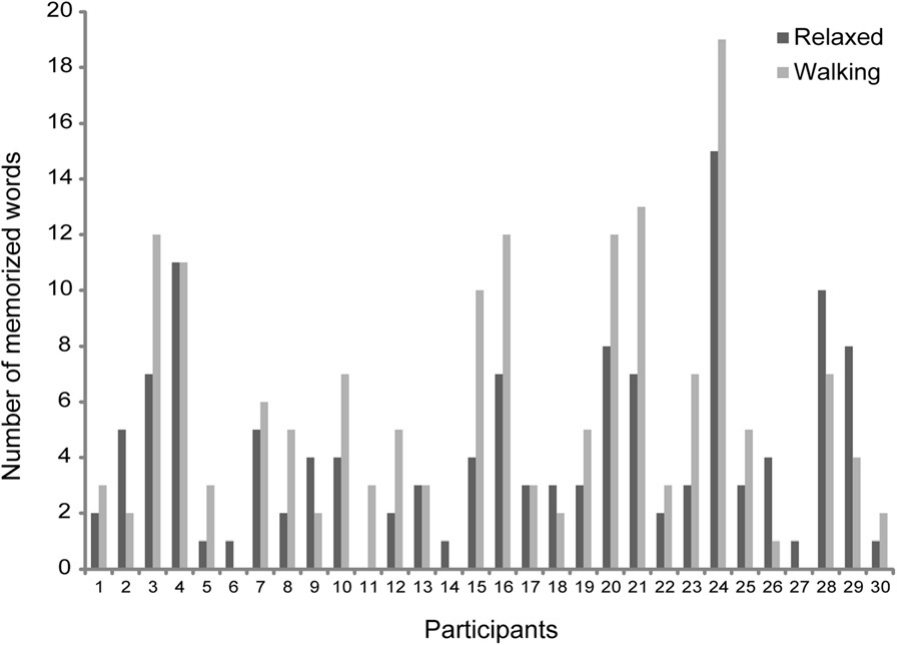
**Vocabulary test performance for each participant in Experiment 2.** Relaxed = dark grey; Walking = light grey.

For Experiment 2, the omnibus ANCOVA revealed a main effect of session (see Figure 1; F (1,28) = 6.44, p = .02, η_p_^2^ = .187; mean treadmill walking: 5.3 (SD: 4.6) words; sedentary: 4.1 (SD: 3.5) words). There were no further interactions. Single subjects’ performance is plotted in Figures 2 and 3, showing that 63% of the participants performed better in the walking condition.

### Serum BDNF & performance

BDNF in serum concentration ranged between 24767 and 53158 pg/ml (mean: 35301 pg/ml; SD: 6799). BDNF values at baseline were 36822 pg/ml (SD: 5645) for the relaxed and 33071 pg/ml (7355) for the walking condition. Post-intervention BDNF values were 31999 pg/ml (SD: 8697) for the relaxed condition and 37531 pg/ml (SD: 7464) for the walking condition. The ANCOVA revealed that BDNF values for male participants were on average higher than for women (F (1,8) = 6.2; p < .05; η_p_^2^ = .436; male: 38598 pg/ml, SD: 8477); female: 37310 pg/ml, SD: 6071). There were no further main effects or interactions. None of the computed correlations between BDNF in serum and vocabulary test performance reached significance.

### Salivary cortisol & performance

**Figure 4:**
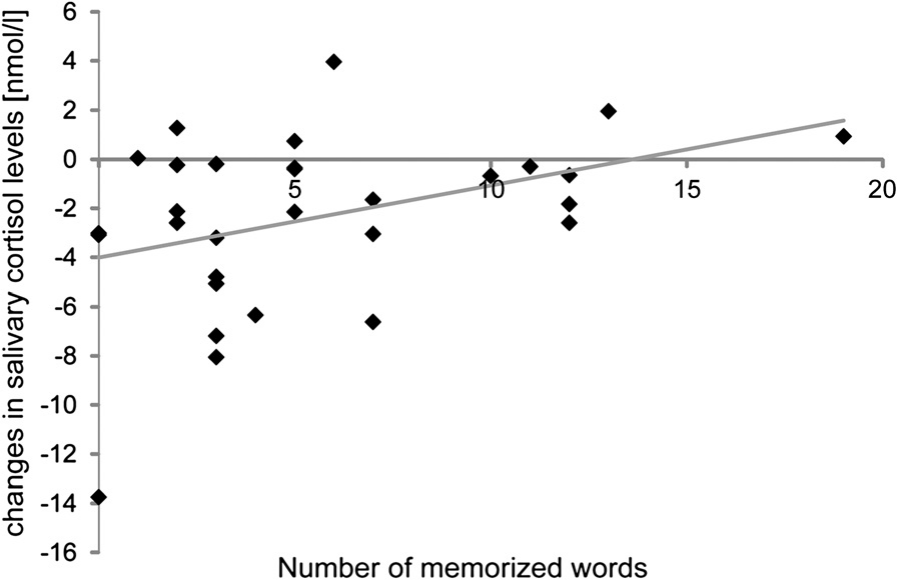
Correlation between changes in cortisol levels during the treadmill-walking learning session and vocabulary test performance.

Cortisol concentrations ranged between 1.3 and 33.8 nmol/l (mean: 27.6 nmol/l; SD: 6.6). Cortisol at baseline was 9.9 (SD: 8.2) nmol/l for the relaxed condition and 10.6 (SD: 7.4) nmol/l for the walking condition. Post-intervention, cortisol was 8.3 nmol/l (6.5) for the relaxed condition and 8.7 (6.5) nmol/l for the walking condition. The ANCOVA on cortisol concentrations did not reveal any significant effects (p > .08). However, there was a positive correlation between changes in cortisol concentration during the treadmill session (Mean: -2.37 nmol/l; SD: 3.52) and performance in the vocabulary test (see Figure 4; r = .386; p = .02) indicating that better performance was associated with a lesser decrease of salivary cortisol. When additionally eliminating the extreme value around -14 nmol/l, the correlation was still significant (r = .322; p = .05).

## Discussion

The current study compared the effects of treadmill walking and physical inactivity on vocabulary learning. Serum BDNF and salivary cortisol concentrations were measured in subgroups of participants to assess the involvement of these substances in the relationship between physical activity and learning.

In both experiments we found that very light treadmill walking during encoding resulted in better performance during vocabulary retrieval compared to being sedentary. In Experiment 1 we found an interaction of day of session x perceived safety on the treadmill showing that participants performed better if they felt safe on the treadmill. However, we did not replicate this effect in Experiment 2. In contrast to our previous studies [20,22] we currently tested equal numbers of female and male participants. We found no interaction involving the factor sex, indicating that the positive effect of walking on verbal long-term memory is independent of sex. One may argue that the effect is not driven by the concurrent motor activity but may also have been observed if the walking task had been performed before the learning task. However, data from our previous study on moderate ergometer cycling [20] have shown that a pre-encoding exercise condition does not improve performance compared to a sedentary condition. Since movement intensity was below 50% of the maximum oxygen consumption in the current series of experiments, we doubt that such a low-intensity motor condition prior to encoding would affect encoding when a moderate exercise bout has no effect. We thus interpret the current results as evidence for a beneficial effect of concurrent motor activity on verbal learning.

On a critical note, one potential limitation of the current study is that participants did not complete an immediate recall test at the end of each experimental session. We deliberately omitted this test to keep the present study comparable to previous ones, where performance was not tested immediately after the encoding phase either [20,22]. However in a recent, not yet published study, we have implemented this additional test. Here motor activity had an even stronger positive effect on retention performance. This evidence suggests that an additional test at the end of each session would most likely have strengthened the current findings.

Concerning the collected endocrinological parameters we found no effect of serum BDNF but of salivary cortisol on vocabulary learning. The results concerning BDNF are in line with our previous findings that serum BDNF is unaffected by moderate-intensity exercise [20,29] and thus does not seem to mediate the described behavioral effect. However they are in contrast to other studies reporting a positive relation between BDNF and memory performance (e.g., [24,25]). On a critical note, it may be that the sampling time was responsible for the negative results. Here the post-intervention sample was taken immediately after the respective intervention had finished which may have been too late to pick up an activity-related increase. However, even when taking blood samples during exercise, BDNF was not enhanced during moderate but only during high-intensity exercise [29]. Thus, we are confident that our reported null effect is not based on a sampling time confound but physiologically real.

Our current results indicate that salivary cortisol might be a mediator variable in the interplay of motor activity and long-term memory. We found that changes in cortisol levels correlated positively with performance in the walking condition. Descriptively, salivary cortisol decreased during treadmill walking which is in contrast to our initial hypotheses. However, this finding indicates that very light treadmill walking is not stressful. This is in line with the missing interaction of the factors safety on the treadmill and session in Experiment 2. Participants in Experiment 2 obviously felt very confident on the treadmill, which might explain why cortisol levels did not increase. On the other hand, significant differences in heart rate between the sedentary and the walking condition suggest that arousal was elevated in the walking condition. However, we found no significant correlations between heart rate and vocabulary test performance.

Taken together, the investigated endocrinological parameters can partly account for the behavioral effect. Our results are in line with previous studies on the effects of glucocorticoids on memory showing that increased cortisol levels improve memory formation, in particular memory for emotionally arousing events (e.g., [50]; for a review, see [51]). As our intervention, i.e. treadmill walking, did not increase cortisol levels, we found that more stable cortisol levels were associated with better memory formation for the present, emotionally neutral stimuli. First, future research needs to assess whether this effect would be stronger if participants were asked to walk faster, i.e. if we increased the physical arousal, or whether this would have the opposite effect. In their seminal work, Kirschbaum et al. [52] investigated the effect of stress on declarative memory (a wordlist recall test). The authors induced stress by using the Trier Social Stress Test [53] and found a strong negative correlation between the cortisol response and memory test performance. Hence, future studies on the effect of exercise-induced cortisol responses and memory need to determine the peak of the inverted U-shape function where exercise intensity becomes too high to increase retention performance. Second, it is an open issue whether emotionally arousing stimuli result in even stronger effects or whether physical arousal affects emotional and neutral stimuli equally. Possibly the previously often used cold pressor stress [50] to increase arousal at initial encoding is specifically effective for emotionally arousing stimuli while exercise as a form of pleasant arousal also affects neutral stimuli. Hence, the specific mechanisms underlying the observed benefits of walking for vocabulary learning remain to be elucidated. Our present results indicate an involvement of the hypothalamus-pituitary adrenal (HPA) axis, which needs to be investigated in more detail.

At least one alternative explanation that we aim to assess in our future experiments is the effect of auditory-motor coupling on retention performance: comparing the present design with studies reporting interference effects for combined walking and learning [15-18], the latter studies used more challenging motor tasks and did not temporally align motor and cognitive activities. In the current study, stimuli were presented at the same rate as the participants’ preferred walking speed. Hence, temporal alignment of stimulus presentation and motor activity may have been critical for the improved performance. This hypothesis would be in line with an entrainment theory where synchronization of internal attending activity with an external event is a prerequisite of attentive processing [54]. Hence, stimulus temporal predictability facilitates the focusing of attention on anticipated points in time, resulting in a more efficient allocation of cognitive resources. However, this hypothesis still needs to be tested. In a currently running study on vocabulary encoding during walking we therefore included the factor timing, i.e. we aim to compare retention performance to temporally aligned versus random vocabulary presentation. Furthermore, recent data from our lab have provided evidence that synchronized motor activity enhances attention allocation [21] which is a prerequisite for learning. Hence, future studies are needed to shed more light on this issue. At the moment we can conclude that slow walking at very light intensity during vocabulary encoding improves verbal long-term memory. The exact mechanism behind this phenomenon remains to be identified by future research.

## Competing interest

The authors declare that they have no competing interest.

## Authors' contribution

Conceived and designed the experiments: MSK, JK. Performed the experiments: NZ, JM. Analyzed the data: MSK, NZ, JM. Contributed materials/analysis tools: CT, LV, CA. Wrote the paper: MSK, JK. All authors read and approved the final manuscript.
